# Dental Pathology and Surgery

**Published:** 1882-05

**Authors:** A. Cornwall


### DENTAL PATHOLOGY AND SURGERY.

BY A. CORNWALL.

As a member of your committee on dental surgery, I desire to call your attention to a few thoughts suggested by the evidences that are presented in our daily practice; of a lack of sufficient conservatism in our profession; of a readiness to yield to the conclusions of an unfavorable prognosis, and shirk labor and responsibility in doubtful cases by a resort to the forceps, in the fear of a failure if we try to save, and a belief that the patient will never know that any other treatment was possible—a desire to do "thorough work"—construing that phrase to mean putting our patients' mouths in such a condition that they are not likely to require our professional services again soon, by making permanent stoppings where such can assuredly be made, and extracting all that there is much doubt about, including all crownless roots.

I think the best practice tends towards the other extreme.

We should not take counsel of our fears, but rather of our hopes; for if we succeed in making a doubtful tooth useful, we have conferred a lasting benefit on our patient; if we fail, the radical remedy can always be resorted to whenever that failure is fully manifest.

Keep the bones together as long as possible.

Especially try to do this for our *young* patient during that critical creative or growing period, extending to the eighteenth or twentieth year.

That is the time that taxes our best judgment and skill.

After that, nature seems to rest from her labors in extending and expanding the osseous frame and confines her efforts to consolidating and strengthening the structure already reared.

The teeth that have safely stood their trials up to that period, with ordinary care, are not likely to cause much trouble afterward. The most of the cavities that make their appearance at a later date could be found in their incipiency at that time, if a sufficiently critical examination were made. The enamel that at twenty is free from crack or disintegration is likely to remain so to the end.

The reason why the human family suffers from dental caries more than other animals, and civilized man more than his barbarous brother, is to be found in the boiling pot and the grinding stone. They have usurped the function of the teeth, that of preparing our food for digestion, and have relegated those organs to comparative idleness. For want of that severe labor for which they were intended, that forcible churning, as it were, in their sockets, that is produced by masticating hard substances, the nutritive vessels have taken on a flaccid, indolent habit, that has become hereditary, and the teeth are erupted before their mail of enamel is sufficiently solidified to successfully withstand the assaults of the disintegrating forces that surround them, and which are augmented by this same indolent habit.

It is quite the proper thing to raise our children on oatmeal mush. It supplies the nutrition required by the growing teeth, but it requires no masticating.

To my thinking, the raw or roasted grain would better subserve the purpose.

As we are not likely to return to so primitive a diet, we can have no fears of a falling off in the demand for our professional services.

As I have said, it is the management of these organs through their first decade that demands our greatest care and patience.

If one is unnecessarily lost through us, we have inflicted an injury on our patient that we can never repair. If it is true that nature soon obliterates the evidences of its ever having existed, is it not by checking development, by absorbing and drawing together the adjacent parts, and foreshortening the process, creating a deformity?

Respectable practitioners have held that an unfavorable prognosis justifies the removal of the sixth-year molars. In my judgment they should not be extracted except under the pressure of a present imperative necessity, such as periosteal inflammation of such a character as to preclude the hope of relieving it by any less radical treatment.

For it they can be made to do duty comfortably for a month, there is hope for them to last a year. If they can be made to last a year, there is a *probability* that they can be made to last ten years, and an indefinite time longer; for nature is all the time laboring with us for their salvation.

While we are combating the external erosion, the pulp is at work within, for in ordinary health calcification never stops, and each year the structure becomes thicker and more dense, and less susceptible to the influence of destructive agents, and the *probability* of the continued usefulness of the organ greater.

The recuperative energy sometimes developed in a soft, sensitive and unpromising denture excels the hopes of the most sanguine.

It is not rare to see a tooth built up and doing service at thirty that had been condemned by a dentist before twenty, and only saved from extraction by the timidity of the patient.

If, through the carelessness of the patient, or our inability to do better, a crown is gone and a pulp devitalized, I believe it is the true business to save the root, and prevent, as long as possible, the anatomical changes that are so sure to follow its loss, and which break up the natural antagonism—set the contiguous teeth to staggering in their sockets, and are far-reaching in their deleterious consequences.

Extraction means absorption—displacement of the remaining teeth, a shrinking together of the middle of the countenance, a deepening of the lines, and a premature look of age and care, that the victim little suspects the cause of.

Each year of experience and observation increases my respect for the methods of the English dentists, who, though they are not always as neat in their operations as we sometimes are, are less addicted to the use of the forceps, and love to save the contour of the face by saving the roots where the crowns are gone, and placing their artificial work on the top of them. The extraction of a good firm root without a crown is often as great a loss to the patient as was the breaking away of the crown.

It was preserving perfectly the symmetrical proportions of the parts, and furnishing the very best possible support for a porcelain or metal crown that would look better, do more service, and give less annoyance than any other kind of work.

There must have been very poor work done in this line many years ago, for there is an unjust and unreasonable prejudice existing against what are called "pivot teeth."

Call it only "setting a porcelain crown," and you evade the power of that prejudice. I believe we should not weary in our efforts to save all we can of the natural person.

I had under examination, but a few days ago, a patient whose teeth at the age of thirteen were so badly decayed, so fragile and tender, nearly every one of them being more or less defective, and presenting such a discouraging pathological condition that the dentist who had charge of the case up to that time (and he was a gentleman of considerable repute in our profession) advised the parents that it was hardly worth while to spend more money on so unpromising a prospect, but to let them go; and as they became too troublesome have them extracted.

A different course was pursued. They were carefully filled, mostly with plastic stoppings, no attempt made at thorough work, none of the sensitive dentine removed more than was absolutely necessary in order to get a place that would hold a covering for it.

They were nursed along with almost constant attention for five years, during which time gutta-percha, gold, tin, amalgam and oxychlorides were tried, gold certainly not giving the best results, operator and patient sometimes becoming nearly discouraged. At the end of that time they began to show a better tone, and at the age of twenty-two the last of them were substantially filled, all but two or three with gold, and they have hardly required any attention since.

Now, at the age of twenty-nine, there are twenty-nine teeth in place, all doing good service, only one of which has had the nerve devitalized, and a good prospect of their remaining comfortable, useful and ornamental to their possessor until old age.

I presume many of you, gentlemen, are reminded of similar cases in your own practice.

I was much interested at our session last year, in the history of a case related by our worthy President, Dr. Younger. He successfully treated a case of salivary calculus, where the process around the lower anterior teeth was nearly all destroyed, so that the teeth would hardly keep an upright position.

It illustrated the readiness of nature to second our efforts at restoration, even in old age.

When we look back to the not very remote period when dentistry was but a very minor branch of surgery, whose representative implements were the turnkey and cautery, and trace its rise into a distinct, large and respectable profession, employing the best efforts of such able and cultivated gentlemen as I see around me to-day, we see the advances it has made in usefulness and dignity, have kept even step with our increasing ability to save and restore.

Much has been accomplished, but I believe the most intelligent and skilled are not satisfied with what they have attained. Let us all strive to reach a higher level.

DR. McLAIN. Mr. President, the subject of the sixth-year molar can not be overestimated. I think when the molar shows that it is past redemption, that it should be extracted at an early day, before the twelfth year. And in that case the second molar making its appearance will naturally take its place, otherwise it would have to be removed at a later period of life, and then it will be apt to produce the effect just given.

THE PRESIDENT. The only trouble in extracting the sixth-year molar, is that the second molars seldom reach the elevation the sixth-year molars occupy, and in that way the lower jaw is made to protrude, and the lower incisors to press on the inner surfaces of the upper incisors. I like to retain the first molars, even under the worst circumstances, until such time as the second molars are fixed in their position, so that they will keep the jaws in proper relation, and prevent the lower incisors from pressing on the upper. The evil of the extraction of the molars of which so little is thought by so many worthy members, is this: that of throwing the lower jaw forward, and either protruding the upper teeth or cutting through the enamel surface and rendering the upper incisors sensitive. Another trouble about extracting the lower molars, and a very considerable one, is this: they possess the largest masticating surface of any teeth, and if extracted you lose the masticating ability of about one-fourth of one side of the jaw.

DR. CORNWALL. In regard to the pressing of the lower incisors on the superior ones, to which you refer, and that protruding of the jaw, there is, in fact, a recession of the jaw caused by the extracting. By the fact of these molars being extracted, all of the teeth have been drawn back and the arch has been protected, so that your lower teeth strike more inside. If the others retain their places, they still hold the lower jaw in form.—*Transactions Cal. State Dental Association.*

